# Ginkgolic acid promotes inflammation and macrophage apoptosis *via* SUMOylation and NF-κB pathways in sepsis

**DOI:** 10.3389/fmed.2022.1108882

**Published:** 2023-01-19

**Authors:** Xinyong Liu, Longwang Chen, Chen Zhang, Wei Dong, Hongbing Liu, Zhong Xiao, Kang Wang, Yaolu Zhang, Yahui Tang, Guangliang Hong, Zhongqiu Lu, Guangju Zhao

**Affiliations:** ^1^Department of Emergency Medicine, The First Affiliated Hospital of Wenzhou Medical University, Wenzhou, China; ^2^Wenzhou Key Laboratory of Emergency and Disaster Medicine, Wenzhou, China

**Keywords:** ginkgolic acid, sepsis, macrophage, SUMO, inflammation, NF-κB

## Abstract

**Background:**

Excessive inflammation and increased apoptosis of macrophages contribute to organ damage and poor prognosis of sepsis. Ginkgolic acid (GA) is a natural constituent extracted from the leaves of *Ginkgo biloba*, that can regulate inflammation and apoptosis. The present study aims to investigate the potential effect of GA in treating sepsis and its possible mechanisms.

**Materials and methods:**

Here, a classic septic mice model and a lipopolysaccharide (LPS)-induced RAW 264.7 inflammation model were established. Cytokines in serum and culture supernatant were detected by ELISA, and the mRNA levels of them were examined by PCR. Hematoxylin and eosin (H&E) staining was performed to determine histopathological changes in liver, lung and kidney. Bacterial burden in the blood, peritoneal lavage fluids (PLFs) and organs were observed on Luria-Bertani agar medium. Flow cytometry and western blotting was used to detect apoptosis and the expression level of apoptosis related molecules, respectively. Moreover, the levels of SUMOylation were detected by western blotting. The activity of NF-κB p65 was assessed by immunofluorescence staining and western blotting.

**Results:**

The result showed that GA promoted inflammatory responses, reduced bacterial clearance, aggravated organ damage, and increased mortality in septic mice. GA increased apoptosis in peritoneal macrophages (PMs) and RAW 264.7 cells. Meanwhile, GA inhibited SUMOylation and increased the nuclear translocation of NF-κB p65 as well as its phosphorylation level.

**Conclusion:**

Collectively, GA promotes inflammation and macrophage apoptosis in sepsis, which may be mediated by inhibiting the SUMOylation process and increasing NF-κB p65 activity.

## Introduction

Sepsis is a syndrome characterized by an abnormal response of the organism to invasive pathogens, which involves alterations in the immune function and leads to multiple life-threatening organ dysfunctions (1). Based on data obtained from developed countries, sepsis impacts 48.9 million patients worldwide each year, and about 20% of deaths are thought to be related to sepsis, severely affecting the quality of life and safety of patients (2, 3). In China, patients with sepsis account for 1/5 of the number admitted to intensive care units, with a 90-days mortality rate of 35.5% (4). Despite many studies on pathogenesis that have been conducted in recent years, which have greatly contributed to the organ support therapy, sepsis remains the major cause of death in critically ill patients (5).

Persistent infection and inflammation are associated with poor prognosis of sepsis (6). In sepsis, macrophages are overactivated and release a large number of pro-inflammatory cytokines, including IL-1β, IL-6, TNF-α, etc., leading to organ damage and death (7, 8). Previous studies found that the excessive inflammation may contribute to immune suppression in sepsis by inducing the apoptosis of immune cells (9, 10). In fact, inhibiting macrophage inflammation and reducing its apoptosis has been proved to reduce organ damage in sepsis and improve survival (11, 12). Nuclear factor-kappaB (NF-κB) is the core regulator of immune response. Once NF-κB is activated, the secretions of a large number of inflammatory cytokines will aggravate secondary damage and induce cell apoptosis in sepsis (13, 14). In many disease models, inhibition of NF-κB activation also has been shown to effectively reduce cell apoptosis (8, 13, 15–17). Therefore, NF-κB is considered as an important therapeutic target for sepsis and inflammatory diseases.

The leaves of *Ginkgo biloba* were used in ancient Chinese medicine to treat lung and cardiovascular disorders. Ginkgolic acid (GA) is a natural constituent extracted from the leaves of *Ginkgo biloba*, *nuts*, and *seed coat*, which has been used as a supplement to traditional Chinese medicine (18). GA is a SUMOylation inhibitor that can inhibit SUMOylation by blocking the formation of E1-SUMO thioester complexes (19, 20). Current studies have reported that the biological activities of GA include anti-tumor, antiviral, neuroprotective, and anti-fibrotic effects, while the ability to modulate immune responses (21–25). However, the effect of GA on sepsis remains unclear and understudied. In evaluating novel therapeutic agents for clinical treatment, it is crucial to determine their effect and specific mechanisms, which can help provide new evidence for the selection of clinical agents in sepsis.

## Materials and methods

### Drugs

Ginkgolic acid (15:1) (purity > 99.9%) was purchased from MedChemExpress (MCE, Shanghai, China). GA was soluble in the dimethyl sulfoxide (DMSO, Solarbio, Beijing, China) for the final concentration of 10 nM for cellular experiments, stored at −80°C. Alternatively, GA was dissolved in 10% DMSO and 90% corn oil for the final concentration of 20 mg/mL for animal experiments.

### Animals

Male C57BL/6N mice (8 weeks old, 22–25 g) were obtained from Shanghai Slack Laboratory Animal Center [license: SCXK (HU) 2012-0002]. Mice were raised in a particular environment with specific pathogen-free conditions under 12 h light/dark cycle, at 22 ± 2°C and appropriate humidity, and had free access to standard chow and water. All experimental procedures and animal welfare protocols complied with the guidelines of the National Institutes of Health Guide for the Care and Use of Laboratory Animals. The methods were reviewed and approved by the Institutional Animal Ethics Committee of Wenzhou Medical University.

### Induction of animal model of sepsis and treatment with GA

Induction of cecal ligation and puncture (CLP) was performed as described previously (26). In short, mice were anesthetized with 1% pentobarbital sodium (50 mg/kg) (TOPNUO, Taizhou, China) injected intraperitoneally. The abdominal skin of the mice was removed, and laparotomy was performed along the mid-abdominal line to separate the cecum, which was ligated at 0.5 cm distal to the cecum tip with a 4-0 non-absorbable suture. The cecum on the ligated side was penetrated with an 18-gauge needle and squeezed with ophthalmic forceps to expel some cecum contents. Then the cecum and feces were returned to the abdominal cavity. The peritoneum and skin were sutured layer by layer and then disinfected with 75% alcohol. Mice were injected subcutaneously with 1 mL of sterile pre-warmed saline behind the neck for fluid resuscitation, and then put back into their cages until recovery.

Mice were randomly divided into five groups: (1) Sham group, (2) CLP group, (3) CLP + GA (10 mg/kg) group, (4) CLP + GA (20 mg/kg) group, (5) CLP + GA (40 mg/kg) group. A single dose of GA (10, 20, or 40 mg/kg body weight) or vehicle (corn oil) was given to mice by gavage 2 h preceding CLP operation. The Sham group was operated as the CLP group, but only the cecum was evacuated and retrieved without ligation or puncture. Mice were then anesthetized and executed at 12, 24, and 48 h after the surgery. The serum and tissue samples were collected for subsequent analyses. For survival analysis, 50 mice were randomized into the Sham, CLP, CLP + GA (10 mg/kg), CLP + GA (20 mg/kg), and CLP + GA (40 mg/kg) groups. Mice were monitored hourly, and their survival was analyzed after 72 h.

### Histological analysis

Mice were sacrificed by carbon dioxide inhalation at 12, 24, and 48 h post-Sham or post-CLP operations. Lung, liver and kidney tissue samples were fixed in 4% paraformaldehyde (Solarbio, Beijing, China) for 48 h at room temperature. After dehydration, clearing, waxing and embedding, the tissue samples were cut into 4 μm-thick sections. According to the manufacturer’s instructions, the paraffin sections were deparaffinized with xylene and stained with hematoxylin and eosin (H&E) (Solarbio, Beijing, China) and followed by observation under an optical microscope.

Lung injury scores were performed as previously described in a double-blinded manner according to neutrophil migration infiltration, hemorrhage and alveolar cavity congestion, and interstitial lung edema (27). The sum of individual scores graded from 0 (normal) to 3 (severe), ranging from 0 to 12. Liver injury score criteria: hepatocyte degeneration or necrosis, hemorrhage or congestion of live tissue, and infiltration of interstitial inflammatory cells. The severity of liver injury was classified as 0, no injury; 1, mild injury; 2, moderate injury; and 3, severe injury (28). The total liver score ranged from 0 to 4. Kidney dysfunction was assessed by measuring tubular morphology. Renal tubular injury was defined as tubular epithelial swelling, brush border detachment, vacuolization, cell necrosis, tubular formation and desquamation. The degree of renal injury was determined depending on the percentage of damaged tubules with the following criteria (29): 0, no injury; 1, < 10% injury; 2, 10–25% injury; 3, 25–50% injury; 4, 50–75% injury; and 5, > 75% injury. The sum of kidney scores ranged from 0 to 5.

### Detection of bacterial burden

The following operations were performed under aseptic conditions. The same volume of whole blood was taken into 100 μL of sodium heparin. The peritoneal lavage fluids (PLFs) were obtained by flushing the peritoneal cavity with 5 mL saline. The lung, liver, and kidney homogenates were derived by grinding in saline. Serial dilutions of blood, PLFs, and tissue homogenates of the lung, liver, and kidney were plated on Luria-Bertani agar medium at 37°C for 24 h. Colony-forming unit (CFU) counts were then counted and calculated.

### Isolation of peritoneal macrophages (PMs)

Peritoneal macrophages (PMs) were isolated from mice as previously described (30). Briefly, a 1.5% soluble starch solution (3 mL/mice, Solarbio, Beijing, China) was injected intraperitoneally into the mice. After 48–72 h, the mice were lavaged intraperitoneally with 10 mL of pre-chilled Roswell Park Memorial Institute (RPMI) 1640 medium (Gibco, NY, USA), divided into three instillations of 3 mL. The recovered medium was centrifugated at 500 *g* for 5 min at 4°C. The suspension with PMs was obtained by removing the red blood cells with RBC lysis buffer (Solarbio, Beijing, China), and washed twice. The precipitate was resuspended in 3 mL of RPMI 1640 medium supplemented with 10% fetal bovine serum (FBS, Gibco, NY, USA), 100 U/mL penicillin, and 100 mg/mL streptomycin. The cells were plated in 6-well plated (2 × 10^6^ cells/well), and cultured at 37°C under a 5% CO_2_ humidified atmosphere for 2 h. The supernatant was removed, and the adherent cells were the PMs.

### Cell culture and treatment

Raw 264.7 cells were purchased from the cell Bank of Shanghai Institutes for Biological Science (Shanghai, China) and cultured in high-glucose Dulbecco’s Modified Eagle’s Medium (DMEM, Gibco, USA) supplemented with 10% fetal bovine serum (FBS, Gibco, USA) at 37°C under a 5% CO_2_ humidified atmosphere. Cells were incubated in six-well plates at a density of 2 × 10^6^ cells/mL and cultured to 80% area. Cells were randomly divided into four groups: Ctrl, GA, LPS, and LPS + GA groups. Then the cells were pretreated with GA (10 nM) for 30 min before lipopolysaccharide (LPS) (1 μg/mL) stimulation for 24 h to establish an inflammation model. The Ctrl group was given the same volume of vehicle (DMOS). The cells and supernatant were collected for the following analysis.

### Quantitative real-time polymerase chain reaction (RT-PCR)

Total RNA in PMs and RAW 264.7 cells was extracted using RNA simple total RNA kit (Tiangen, Beijing, China) according to the manufacturer’s instructions. cDNA was synthesized from quantified RNA by using ReverAid RT kit (Thermo Fisher, MA, USA). The target mRNAs were quantified by Q-PCR using SYBR Green Master Mix (Bio-rad, CA, USA) on a BioRad CFX96 (Bio-rad, CA, USA). The amplification curves and lysis curves were confirmed to be normal in the results after the reaction, and the relative quantitative expression of the target gene was calculated with 2^–ΔΔ*CT*^ values using GAPDH as the internal reference gene. Mouse primer sequences are shown in Table 1.

**TABLE 1:** List of primer sequences.

Primer name		Primer sequences
GAPDH	Forward primer:	5-CAATGCATCCTGCACCACCA-3
	Reverse primer:	5-CACGCCACAGCTTTCCAGAG-3
IL-1β	Forward primer:	5-CACTACAGGCTCCGAGATGAACAAC-3
	Reverse primer:	5-TGTCGTTGCTTGGTTCTCCTTGTAC-3
IL-6	Forward primer:	5-CTTCTTGGGACTGATGCTGGTGAC-3
	Reverse primer:	5-TCTGTTGGGAGTGGTATCCTCTGTG-3
TNF-α	Forward primer:	5-CGCTCTTCTGTCTACTGAACTTCGG-3
	Reverse primer:	5-GTGGTTTGTGAGTGTGAGGGTCTG-3
IL-10	Forward primer:	5-AGAGAAGCATGGCCCAGAAATCAAG-3
	Reverse primer:	5-CTTCACCTGCTCCACTGCCTTG-3
iNOS	Forward primer:	5-ATCTTGGAGCGAGTTGTGGATTGTC-3
	Reverse primer:	5-TAGGTGAGGGCTTGGCTGAGTG-3

### Enzyme-linked immunosorbent assay (ELISA)

Mice were anesthetized, and blood was obtained aseptically. After agglutinating for 60 min at room temperature, serum was collected by centrifugation at 1,000 *g* for 10 min at 4°C. The serum was dispensed and stored at −80°C for storage. The culture supernatants of RAW 264.7 cell were collected after stimulation with 1 μg/mL LPS for 24 h. The levels of Interleukin (IL)-1β, IL-6, and tumor necrosis factors (TNF)-α in the serum and the culture supernatants were detected using ELISA kits (Multisciences, Hangzhou, China).

### Immunofluorescence staining

RAW 264.7 cells were plated in a 6-well plate (10^4^ cells/well) containing cell crawlers, shaken and walled, and then grouped for treatment. After 6 h of cell culture, cells were fixed with 4% paraformaldehyde for 15 min and permeabilized with PBS containing 0.3% TritonX-100 for 20 min. PBS blocking solution containing 1% BSA was added to block the cells for 1 h. The primary antibody, rabbit anti-p-NF-κB p65 antibody (1:1000 dilution, CST, USA), was incubated overnight at 4°C. Washed twice, cells were incubated with the secondary antibody (1:1000 dilution, Abcam, Cambridge, UK) in the dark at room temperature for 1 h. The nuclei were stained with 4’,6-diamidino-2-phenylindole (DAPI) for 5 min. The samples were exposed to anti-fluorescence quenching sealing solution (Solarbio, Beijing, China). A laser confocal microscope was used to observe under the corresponding excitation light, and images were acquired.

### Flow cytometry

Apoptosis of cells was detected with the fluorescent probe FITC-labeled Annexin V, Annexin V-FITC. Every 10^6^ cells were collected and washed twice with pro-chilled PBS, resuspended in 500 μL Binding Buffer. The cells were incubated with 5 μL Annexin V- FITC and 10 μL PI for 5 min at room temperature for flow cytometry analysis, protected from light. The results obtained were analyzed by FlowJo software (Ashland, USA).

### Western blotting analysis

The cells were collected and washed with PBS, lysed in radioimmunoprecipitation (RIPA) lysis buffer (Solarbio, Beijing, China) with 1% phenylmethylsulfonyl fluoride (PMSF) (Solarbio, Beijing, China) and 1% protease and phosphatase inhibitor (Solarbio, Beijing, China) on ice for 30 min. The supernatant was obtained by centrifuging at 13,000 *g* for 20 min at 4°C, followed by quantification of the protein concentration with a bicinchoninic acid (BCA) kit (Thermo Fisher, MA, USA). Then the protein was mixed with 5 × protein loading buffer (Thermo Fisher, MA, USA) and denatured at 100°C for 10 min. Equal amounts of denatured proteins (30 μg) were separated on 10% sodium dodecyl sulfate (SDS)-polyacrylamide gel electrophoresis (PAGE) (Beyotime, Shanghai, China) by western blot and transferred onto 0.45 μm polyvinylidene fluoride (PVDF) membranes (Millipore, CA, USA) for immunoblotting; Next, the membranes were incubated with 5% fat-free dry milk in Tris-buffered saline containing Tween-20 (TBST) (Solarbio, Beijing, China) for 2 h at room temperature, followed by specific primary antibodies (anti-SUMO1, anti-SUMO2/3, anti-BCL-2, anti-BAX, anti-Cleaved caspase 3, anti-NF-κB p65, anti-phospho-NF-κB p65, anti-β-actin, anti-GAPDH, both 1:1000 dilution) (Abcam, Cambridge, UK) at 4°C overnight. The membranes were washed three times and incubated with the horseradish peroxidase (HRP)-conjugated secondary antibody (1:5000 dilution) (Beyotime, Shanghai, China) for 60 min at room temperature. Finally, protein bands were visualized by using the enhanced chemiluminescence (ECL) kit (Thermo Fisher, MA, USA) and performed with the ChemiDic TM XRS + Imaging System (Hercules, CA, USA). The density of each band was analyzed using Image J software (NIH Image, Bethesda, MD).

### Statistical analysis

Data were analyzed by using GraphPad Prism 9.0 (GraphPad Software, La Jolla, CA, USA). The experiment was repeated at least three times. One-way ANOVA followed by Least Significant Difference (LSD) multiple comparison tests was performed for assessing statistical significance between multiple groups. *P* < 0.05 were considered statistically significant.

## Results

### GA upregulates inflammatory response

**FIGURE 1:**
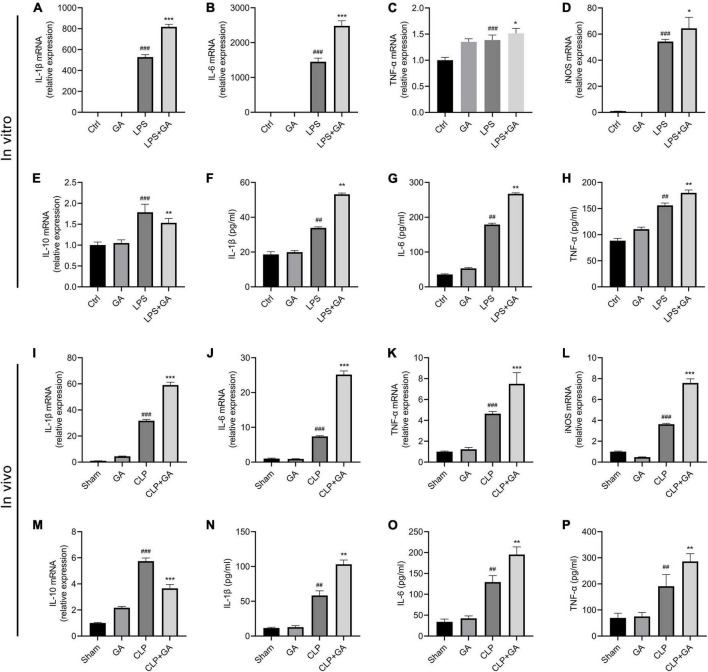
Ginkgolic acid (GA) upregulates inflammatory response *in vitro* and *in vivo*. RAW 264.7 cells and their culture supernatants were collected 24 h after lipopolysaccharide (LPS) stimulation. The mRNA levels of **(A)** IL-1β, **(B)** IL-6, **(C)** TNF-α, **(D)** iNOS, and **(E)** IL-10 were detected by real-time polymerase chain reaction (RT-PCR) (*n* = 6). The protein levels of **(F)** IL-1β, **(G)** IL-6, and **(H)** TNF-α in the cell culture supernatant were detected by enzyme-linked immunosorbent assay (ELISA) (*n* = 3). Data were calculated with the mean ± SD. ^##^*P* < 0.01, ^###^*P* < 0.001 versus the Ctrl group; *P < 0.05, ^**^P < 0.01, ^***^P < 0.001 versus the LPS group. PMs and serum samples were collected from mice (*n* = 4) at 24 h after CLP. The mRNA levels of pro-inflammatory factors **(I)** IL-1β, **(J)** IL-6, **(K)** TNF-α, **(L)** iNOS, and anti-inflammatory factor **(M)** IL-10 in PMs were detected by RT-PCR. The protein levels of **(N)** IL-1β, **(O)** IL-6, and **(P)** TNF-α in serum (*n* = 6) were detected by ELISA. Data were calculated with the mean ± SD. ^##^*P* < 0.01, ^###^*P* < 0.001 versus the Sham group; ^**^P < 0.01, ^***^P < 0.001 versus the CLP group.

Inflammatory response is a highly prevalent symptom of sepsis (31). To determine whether GA affects the inflammatory response, mRNA levels of pro-inflammatory factors IL-1β, IL-6, TNF-α, inducible nitric oxide synthase (iNOS), and anti-inflammatory factor IL-10 were measured in PMs of septic mice and RAW 264.7 cells. In the *in vivo* experiment, mRNA levels of IL-1β, IL-6, TNF-α and iNOS were increased in PMs of mice 24 h after CLP compared to the Sham group, and mRNA levels of IL-10 were also mildly increased (Figures 1A–E). GA treatment further increased the mRNA levels of inflammatory markers, while mRNA levels of IL-10 decreased (Figures 1A–E). Consistent with the *in vivo* experiments, in the LPS-stimulated RAW 264.7 cell inflammation model, GA promoted the mRNA levels of IL-1β, IL-6, TNF-α and iNOS and inhibited the mRNA levels of IL-10 mRNA (Figures 1I–M). In addition, we measured the protein levels of IL-1β, IL-6, and TNF-α in blood samples from septic mice and supernatants of RAW 264.7 cells. The results also showed that GA increased IL-1β, IL-6, and TNF-α levels *in vitro* (Figures 1F–H) and *in vivo* (Figures 1N–P).

### GA aggravates sepsis-induced organ pathological damage and reduces survival rate

**FIGURE 2:**
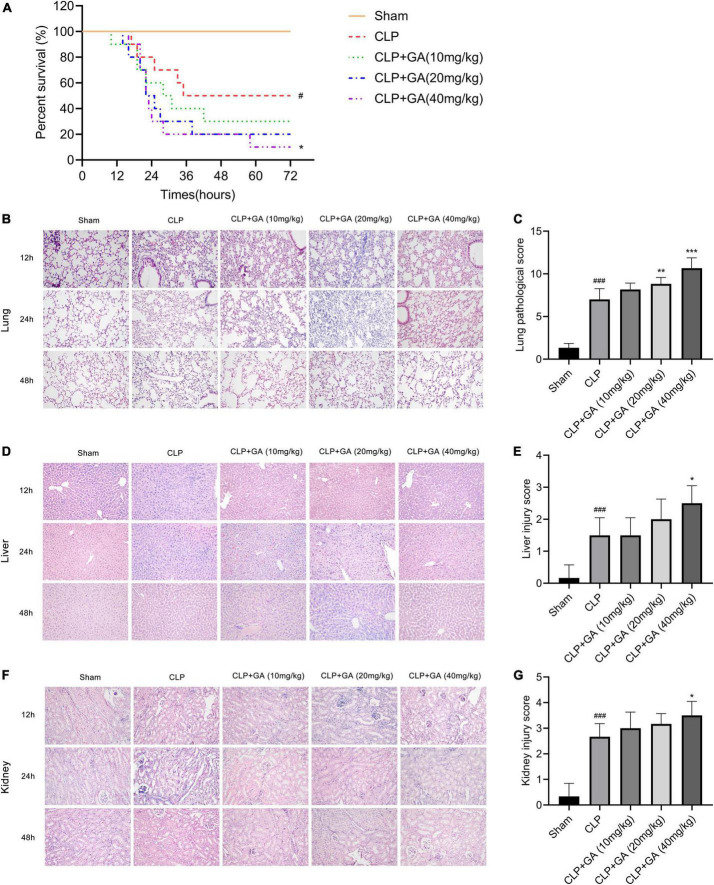
Ginkgolic acid (GA) reduces survival rate and aggravates sepsis-induced organ pathological damage. **(A)** Kaplan-Meier survival curve for mice 72 h after the cecal ligation and puncture (CLP) operation (*n* = 10). The status of the mice was observed every hour and the death of the mice was recorded. Data were calculated with the mean ± SD. ^#^*P* < 0.05 versus the Sham group; *P < 0.05 versus the CLP group. Pathological changes of organs in mice were observed by H&E stain, during CLP-induced sepsis for 12, 24, and 48 h. Representative histopathological characteristics of **(B)** lungs, **(D)** liver, and **(F)** kidneys by H&E staining. Histological scores of CLP mice at 24 h treated with GA or vehicle were shown in **(B,E,G)**. Data were calculated with the mean ± SD (*n* = 6). ^#^*P* < 0.05, ^###^*P* < 0.001 versus the Sham group; *P < 0.05, ^**^P < 0.01, ^***^P < 0.001 versus the CLP group.

Relative to that in the sham group, the 72-h survival rate was markedly reduced in the CLP group, but was further decreased in the GA-treated mice (Figure 2A). Next, the effect of GA on the lung, liver and kidney was assessed by H&E staining during 12, 24, and 48 h of CLP-induced sepsis. Compared to the CLP group, the CLP + GA group exhibited more severe neutrophil migration and infiltration, hemorrhage, alveolar cavity congestion, and interstitial lung edema in a dose-dependent manner (Figures 2B, C). Meanwhile, the liver cell borders were blunted received with GA. The markedly severe edema, inflammatory cell infiltration and hepatocyte necrosis were observed (Figures 2D, E). Further, the mice with GA treatment exhibited more severe renal tubular damage (Figures 2F, G). These indicate that GA exacerbates pathological organ changes in septic mice.

### GA increases bacterial burdens in blood, and PLFs, and organs of septic mice

**FIGURE 3:**
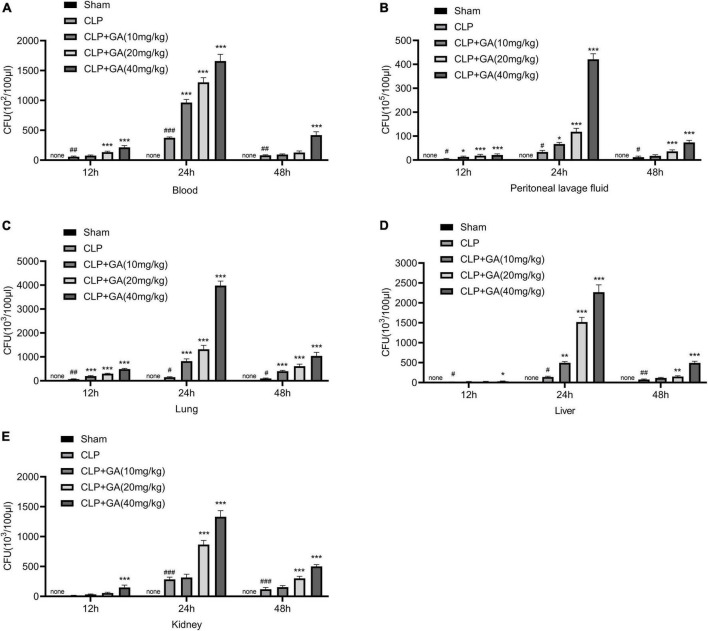
Ginkgolic acid (GA) increases bacterial burdens in blood, peritoneal lavage fluids (PLFs), and organs of septic mice. Blood, PLFs, lungs, liver, and kidneys were collected aseptically at 12, 24, and 48 h after cecal ligation and puncture (CLP) operation. Bacterial colony-forming unit (CFU) counts were performed in **(A)** blood, **(B)** PLFs and tissue homogenates of **(C)** lungs, **(D)** liver, and **(E)** kidneys. CFU indicates colony forming units. Data were calculated with the mean ± SD (*n* = 4). ^#^*P* < 0.05, ^##^*P* < 0.01, ^###^*P* < 0.001 versus the Sham group; *P < 0.05, ^**^P < 0.01, ^***^P < 0.001 versus the CLP group.

To explore the bacterial burdens of mice, blood and PLFs were aseptically collected for 12, 24, and 48 h after surgery, followed by incubation for 24 h. The results showed that the bacterial levels in the blood and PLFs were significantly increased in the CLP group, which was further increased in the mice given GA in a dose-dependent effect (Figures 3A, B). In addition, we also examined the bacterial load in the lungs, liver, and kidneys, and the results were similar to those of blood and PLFs (Figures 3C–E).

### SUMO1 and SUMO2/3 are downregulated in macrophages, and are inhibited by GA *in vivo* and *in vitro*

**FIGURE 4:**
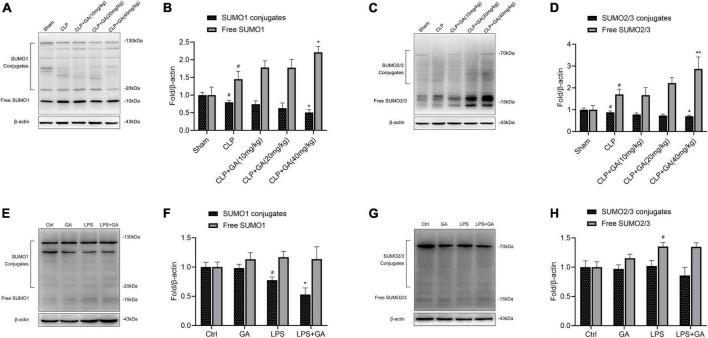
SUMO1 and SUMO2/3 are downregulated in macrophages and are inhibited by ginkgolic acid (GA) *in vivo* and *vitro*. **(A,C)** Representative image of western blotting analysis of the levels of SUMO1 and SUMO2/3 in the peritoneal macrophages (PMs) of mice at 24 h after the cecal ligation and puncture (CLP), treated with different doses of GA. Quantification of western blotting data was shown in **(B,D)**. Data were calculated with the mean ± SD (*n* = 3). ^#^*P* < 0.05 versus the Sham group; *P < 0.05, ^**^P < 0.01 versus the CLP group. **(E,G)** Representative image of western blotting analysis of the levels of SUMO1 and SUMO2/3 in RAW 264.7 cell at 24 h after the stimulation of lipopolysaccharide (LPS), treated with different doses of GA or vehicle. Quantification of western blotting data was shown in **(F,H)**. Data were calculated with the mean ± SD (*n* = 3). ^#^*P* < 0.05 versus the Ctrl group; *P < 0.05 versus the LPS group.

The levels of SUMO1 and SUMO2/3 in macrophage during sepsis and the inhibitory effect of GA were unclear, which were detected by western blotting. Compared with the Sham group, the protein levels of conjugate-SUMO1 and conjugate-SUMO2/3 were reduced in PMs of CLP-operated mice, and the protein levels of free SUMO1 and free SUMO2/3 were increased, which showed that SUMOylation was attenuated. Treating with GA, the level of SUMOylation was significantly lower and showed a dose-dependent effect (Figures 4A–D). In addition, in the LPS-stimulated RAW 264.7 cell inflammation model, the levels of SUMO1 conjugates were also suppressed, which were exacerbated by GA. However, the levels of free SUMO1 and free SUMO2/3 were not increased by GA administration (Figures 4E–H).

### GA promotes NF-κB p65 phosphorylation and nuclear translocation

**FIGURE 5:**
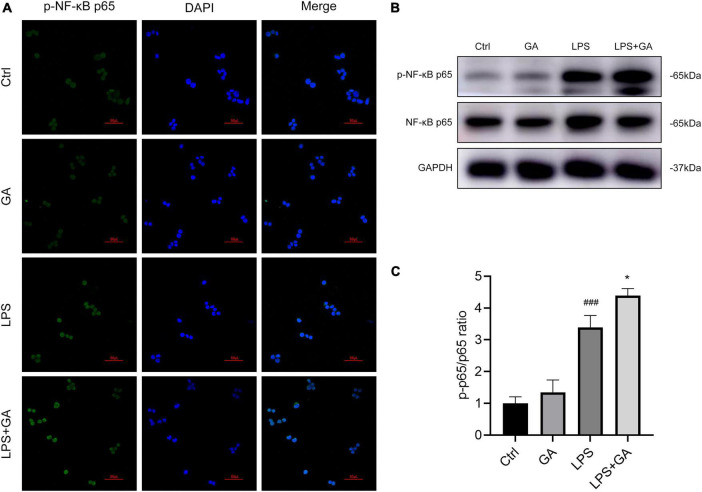
Ginkgolic acid (GA) promotes NF-κB p65 phosphorylation and nuclear translocation. **(A)** The changes of p-NF-κB p65 nuclear translocation in RAW 264.7 cell after lipopolysaccharide (LPS) stimulation for 6 h were detected by immunofluorescence staining, treated with GA or vehicle (*n* = 3). **(B)** Detection of the protein levels of p-NF-κB p65 and NF-κB p65 in RAW 264.7 cell after 6 h of LPS stimulation by western blot (*n* = 3), and p-p65/p65 ratio was shown in **(C)**. ^###^*P* < 0.001 versus the Ctrl group; *P < 0.05 versus the LPS group.

We used immunofluorescence technique to detect the activation of NF-κB p65 into the nucleus of RAW 264.7 cells. As shown in Figure 5A, the nuclear translocation of phosphorylated NF-κB p65 (p-NF-κB p65) was increased after LPS treatment, and the fluorescence intensity of p-NF-κB p65 in the nucleus was increased. Next, we treated the cells with GA. The results showed that the nuclear translocation of p-NF-κB p65 was more pronounced. In addition, we used western blot to detect the protein levels of NF-κB p65, and obtained the same results as for immunofluorescence (Figures 5B, C). Total intracellular p-NF-κB p65 protein content increased after LPS stimulation, and this phenomenon was further aggravated by treatment with GA. Those suggested that GA in the LPS-stimulated model *in vitro* promotes NF-κB p65 phosphorylation and nuclear translocation.

### GA exacerbates macrophage apoptosis

**FIGURE 6:**
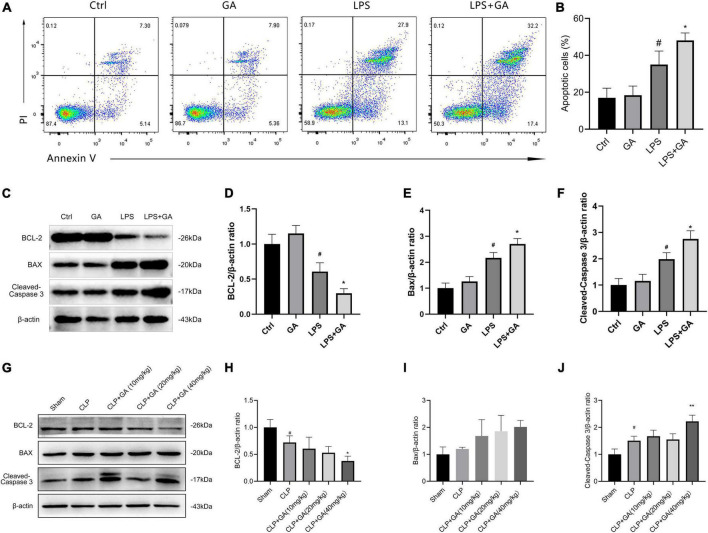
Ginkgolic acid (GA) exacerbates macrophage apoptosis. **(A)** Representative flow cytometric pictures of the percentage of apoptotic cells in RAW 264.7 cell at 24 h after the stimulation of lipopolysaccharide (LPS), treated with GA or vehicle (*n* = 3). Data were showed with the mean ± SD in **(B)**. ^#^*P* < 0.05 versus the Ctrl group; *P < 0.05 versus the LPS group. **(C)** Representative image of western blotting analysis of BCL2, BAX, and Cleaved-Caspase 3 in RAW 264.7 cell at 24 h after the stimulation of LPS, treated with GA or vehicle (*n* = 3). Data were showed with the mean ± SD in **(D–F)**. ^#^*P* < 0.05 versus the Ctrl group; *P < 0.05 versus the LPS group. **(G)** Representative image of western blotting analysis of BCL2, BAX, and Cleaved-Caspase 3 in the PMs of septic mice (*n* = 3). Quantification of western blotting data were shown in **(H–J)**. Data were calculated with the mean ± SD. ^#^*P* < 0.05 versus the Sham group; *P < 0.05, ^**^P < 0.01 versus the CLP group.

We verified the effect of GA on apoptosis in RAW 264.7 cells *in vitro*. The results showed that the LPS group had higher proportion of apoptotic cells compared to Ctrl group. Meanwhile, GA-treated group exhibited more apoptotic cells (Figures 6A, B). Furthermore, the levels of BCL-2, BAX, and Cleaved-Caspase 3 in RAW 264.7 cells were detected by western blot. After LPS stimulation for 24 h, the level of Cleaved-Caspase 3 and BAX were elevated, the level of BCL-2 was decreased (Figures 6C–F). This phenomenon was further aggravated by administration of GA. Moreover, we verified the effect of GA on apoptosis in PMs *in vivo*. The results showed that apoptosis was increased in the CLP group compared to Sham group. Meanwhile, GA-treated group exhibited more severe apoptosis (Figures 6G–J).

## Discussion

Sepsis is characterized by persistent infection and an uncontrolled systemic inflammatory response that leads to tissue damage and ultimately to multisystem organ failure, for which no specific therapeutic agents exist (32, 33). Macrophages are widely distributed in various tissues and play various roles in phagocytosis, bactericidal, antigen presentation, and inflammatory factor secretion in all stages of sepsis. Macrophages secrete large amounts of pro-inflammatory cytokines that aggravate the inflammatory response and lead to organ damage (34, 35). Therefore, study of the effect of therapeutic agents on the pathophysiological process of macrophage injury contributes to the improvement of targeted therapy for sepsis. GA has been shown to have antitumor, antiviral, and antifibrotic properties, but its regulation of the inflammatory response in sepsis is unclear. Our study confirmed that GA modulated the inflammatory response, attenuated bacterial clearance, exacerbated organ damage, and significantly increased mortality in mice. Furthermore, this process may be achieved through inhibiting the SUMOylation process and promoting NF-κB expression.

Cytokines play a key role in the recruitment of leukocytes to inflamed tissues, but they are often described as a “double-edged sword.” The defense response during sepsis requires an appropriate balance of anti-inflammatory and pro-inflammatory cytokines (36, 37). Several reports have shown that TNF-α, IL-6, and IL-10 can be used as criteria for evaluating the severity of sepsis, and that elevated levels of cytokines predict mortality in septic mice (38, 39). Changes in the levels of inflammatory factors in the blood reflect the severity of the systemic inflammatory response (40). The levels of pro-inflammatory cytokines IL-1β, IL-6, and TNF-α in the blood of septic mice were significantly increased after treatment with GA. In addition, in the CLP model, septic mice are first infected in the abdominal cavity, where abdominal bacteria invade the organs and blood, causing local and systemic inflammatory responses (41). Therefore, we observed the expression of inflammatory factors in the PMs of septic mice. The results further confirmed that GA promoted the mRNA levels of pro-inflammatory factors. In addition, we also verified the effect of GA on macrophages *in vitro*, and the results similarly confirmed that GA promoted the mRNA and protein expression of inflammatory factors. Those suggest that GA modulates cytokine homeostasis toward a pro-inflammatory mode in septic mice.

The pathogenesis of sepsis is a dysregulated response to infection, in terms of not only enhanced inflammation but also immunosuppression. This abnormal response to infection leads to cellular dysfunction and ultimately to organ dysfunction (42). We constructed mouse models of sepsis by CLP and pretreated these with different concentrations of GA for 2 h and observed the 72-h survival rate of mice, as well as the degree of organ damage. Consistent with the previous study, the pathological damage to the organs of mice after modeling was aggravated (43, 44). GA exacerbated the mortality of septic mice, accompanied by a significant increase in the concentration of GA. In parallel, GA significantly aggravated the pathological damage of organs such as lungs, liver and kidneys in septic mice, suggesting that GA exacerbates organ dysfunction and contributes to mortality in septic mice during the pathogenesis of sepsis.

Severe bacterial infection is an important factor in the progression of sepsis. Intestinal mucosal barrier dysfunction is essential in sepsis combined with multiple organ dysfunction syndromes (45, 46). Biologically, the intestinal mucosal barrier prevents the entry of intestinal microorganisms, as well as their products, from the intestine into the bloodstream. When sepsis occurs, excessive inflammation damages intestinal epithelial cells, resulting in injury to the intestinal mucosal barrier, which promotes bacterial migration and toxin delivery (47, 48). The bacterial burdens of peripheral blood indicated the severity of systemic infection in mice, while the bacterial burdens in the PLFs could be used as a measure of the severity of abdominal bacterial infection. Our study showed that GA caused an increase in bacterial contents in the blood and PLFs with CLP operation, suggesting that GA reduces the bacterial clearance of the body. Besides, the bacterial clearance capacity of organs is one of the indicators of the risk level of sepsis process. The results showed that the bacterial burdens of the liver, lungs, and kidneys were significantly increased in septic mice with GA administration, and it increased with the higher of drug concentration. This suggests that GA reduces the bacterial clearance capacity of organs, leading to organ dysfunction and aggravating organ damage.

SUMO is a class of ubiquitinated proteins that modifies various proteins that regulate gene expression, including transcription factors, transcriptional cofactors, and factors that regulate chromatin structure (49). SUMO1 mainly modifies proteins in the physiological state. SUMO2 and SUMO3 are very close in amino acid sequence and mainly modify stress proteins (50). Studies have shown that GA impairs SUMOylation by blocking the formation of the E1-SUMO thioester complex (51) and inhibits NF-κB activity (52). To observe whether SUMOylation process is involved in the pathogenesis of sepsis, we used GA to inhibit SUMOylation in septic mice. The results showed that SUMOylation levels were reduced in the PMs, which were further reduced with the administration of GA. *In vitro*, SUMOylation was also inhibited by LPS in RAW 264.7 cell, and GA exhibited a significant SUMOylation inhibitory effect. SUMOylation is characterized by the addition or detachment of SUMO proteins from lysine residues on target proteins, thereby altering their subcellular localization, activity and stability (53). Some evidence indicated that SUMOylation is involved in the NF-κB pathway and plays an important role in inflammation and secretion of various chemokines (54). SUMO competes with ubiquitin for the same binding site of NF-κB inhibitor protein α (IκBα) and inhibits the degradation of IκBα by the ubiquitin proteasome (55). SUMO can bind IκB and inhibit its phosphorylation, hence inhibiting NF-κB in the nucleus to initiate transcription of target genes (56). Thus, SUMOylation and de-SUMOylation play an important role in regulating NF-κB activation and NF-κB-dependent transcription (57, 58). Our results confirm that GA promoted the phosphorylation of NF-κB p65 and its translocation from the cytoplasm to the nucleus, which was speculated to be associated with SUMOylation. Based on the above studies, we determined that GA in RAW 264.7 cells promoted the phosphorylation and nuclear translocation of NF-κB p65. However, the exact effect of SUMOylation inhibition on the phosphorylation of NF-κB p65 is unknown and needs further exploration.

Apoptosis is one of the most important mechanisms of sepsis-induced immunosuppression. Studies have shown that in patients who die from sepsis, increased macrophage apoptosis led to a significant reduction in number in the circulation (59). Our results indicate that GA exacerbates macrophage apoptosis *in vivo* and *vitro*. Meanwhile, excessive inflammatory responses and NF-κB activation cause massive macrophage death and the remaining surviving macrophages *in vivo* are insufficient to clear sepsis-induced bacterial infections (13, 60–62), which may be an important reason for the exacerbation of bacterial infections in the blood and organs of septic mice after GA administration.

Collectively, GA increases macrophage inflammation and apoptosis, and causes organ damage in septic mice. It may be mediated by regulating SUMOylation process and increasing NF-κB p65 phosphorylation with nuclear translocation. In addition, the interaction of SUMOylation on macrophages in sepsis still needs to be further verified using SUMOylation activators.

## Data availability statement

The raw data supporting the conclusions of this article will be made available by the authors, without undue reservation.

## Ethics statement

The animal study was reviewed and approved by the Institutional Animal Ethics Committee of Wenzhou Medical University.

## Author contributions

XL, LC, and ZL contributed to the conception and design of the study. XL, LC, CZ, WD, and HL performed the experiments. ZX, CZ, and KW contributed to the data collection. XL, YZ, CZ, YT, and GH performed the statistical analysis. XL, LC, and GZ drafted the manuscript. ZL and GZ contributed to the final manuscript version and funds collection. All authors approved the final version of the manuscript.
